# Evolution of Ti_3_C_2_ MXene Quantum Dots for Photocatalytic and Photoelectrochemical Applications: A Review

**DOI:** 10.3390/ma19102095

**Published:** 2026-05-16

**Authors:** Adem Sreedhar, Jin-Seo Noh

**Affiliations:** Department of Physics and Semiconductor Science, Gachon University, 1342 Seongnamdae ro, Sujeong gu, Seongnam si, Gyeonggi-do 461-701, Republic of Korea; ademgu@gachon.ac.kr

**Keywords:** Ti_3_C_2_ QDs, surface-active sites, band gap, sustainable environment, stability

## Abstract

The key transformation of 2D Ti_3_C_2_ MXene nanosheets into 0D Ti_3_C_2_ MXene quantum dots (Ti_3_C_2_ QDs) restructures the landscape of surface-active sites and tunable band gaps, enabling visiblelight-driven photocatalytic activity. Interestingly, the evolution of these fascinating Ti_3_C_2_ QDs retains ordered structural characteristics like the parent 2D Ti_3_C_2_ MXene nanosheets with controlled surface chemistry even after the facile hydrothermal process. In particular, evidence of tailoring of Ti_3_C_2_ QDs smaller than 10 nm reinforces the charge carrier separation and suppresses recombination under the strong association of quantum confinement and edge effects. Thus, the physical effects of Ti_3_C_2_ QDs effectively control the limitations of semiconductors, such as charge carrier recombination, slow charge carrier separation, and transportation in the resultant photocatalyst, for the implementation of promising toxic matter degradation and clean H_2_ production. Special considerations are given to the regulation of charge carrier generation and separation for stable photocatalytic performance, such as appropriate band gap formation, localized surface plasmonic behavior, and Schottky barrier formation at the semiconductor interface. Specifically, pure Ti_3_C_2_ QDs with a size smaller than 10 nm exhibit a band gap of 2.16 eV, which has been found to be a powerful way to enable semiconductor-like photoresponse behavior. Overall, the above features make Ti_3_C_2_ QDs the preferred choice for facilitating effective charge carrier dynamics for the optimization of chemical stability in optoelectronic applications. The paper concludes with challenges and future perspectives to guide the 0D Ti_3_C_2_ QDs practical applicability in light-driven and sustainable environmental applications.

## 1. Introduction

The invention of two-dimensional (2D) Ti_3_C_2_ MXene from its three-dimensional (3D) Ti_3_AlC_2_ MAX precursor (selectively removing the Al layer) has significantly advanced applications in the energy, environmental remediation, and safety sectors [1,2,3]. In particular, 2D MXene nanosheets comprising metal carbides, nitrides, and carbonitrides have attracted considerable attention for energy storage and conversion due to their intrinsic properties, including high electrical conductivity, excellent mechanical stability, large surface area, and hydrophilic nature [4]. MXenes are generally represented by the chemical formula M_n+1_X_n_T_x_, where M denotes transition metals (Cr, Ti, Mo, Nb, etc.), X represents C and/or N, and T_x_ refers to surface functional groups (-OH, -O, -F, and -Cl) with n ranging from 1 to 4 [5]. Among various evolved MXene families (M_2_X, M_3×2_, M_4_X_3_, and M_5_X_4_), Ti_3_C_2_ MXene has gained the most widespread attention for diverse applications [6]. Furthermore, the integration of 2D Ti_3_C_2_ MXene nanosheets with semiconductor materials has demonstrated strong potential in photoelectrochemical water splitting [7]. The ability to transform 2D MXene structures into 0D nanostructures, such as nanoparticles or quantum dots, has further expanded their applicability in environmental remediation, due to enhanced surface area and quantum confinement effects [8]. Notably, quantum dots exhibit remarkable efficiency in degrading toxic pollutants under light irradiation [9]. In this context, Ti_3_C_2_ QDs have shown significant promise in toxic matter degradation and photoelectrochemical water splitting. Ti_3_C_2_ MXene quantum dots were first successfully synthesized from 2D Ti_3_C_2_ nanosheets by adopting a hydrothermal process in 2017 [10]. Importantly, the transition of 2D Ti_3_C_2_ MXene nanosheets into 0D QDs preserves the key merits of the parent 2D layer structure. Specifically, the evolution of 0D Ti_3_C_2_ QDs < 10 nm retains key structural features while introducing quantum confinement and edge effects. These effects enhance surface reactivity and facilitate functionalization more effectively than their 2D counterparts [11]. Moreover, MXene QDs with sizes below 10 nm offer significant advantages, including shortened charge transport pathways for facilitating efficient charge transfer during the photocatalytic activity process [12]. Additionally, QDs < 10 nm maintain excellent compatibility with other functional materials, enabling the formation of effective hybrid systems [13]. These characteristics impart distinct electrical and optical properties to Ti_3_C_2_ QDs compared to their 2D MXene nanosheet counterparts [14]. Moreover, the enhanced charge carrier separation (electrons and holes) in QDs is attributed to a high extinction coefficient and dielectric constant, which influence the photocatalytic performance [15]. The above unique structural, optical, and electrical features make Ti_3_C_2_ QDs promising candidates for a wide range of light-active applications. To date, the hydrothermal process [10] and electrochemical etching [16] methods have been primarily employed to produce Ti_3_C_2_ QDs. Interestingly, the transformation of 2D Ti_3_C_2_ MXene into 0D QDs induces semiconductor-like behavior along with increased active sites and a higher surface-to-volume ratio. It is also observed that the characteristic (002) peak of Ti_3_C_2_ QDs moves to a lower angle side compared to 2D Ti_3_C_2_ MXene, indicating changes in interlayer spacing during dimensional reduction [17]. Importantly, incorporating Ti_3_C_2_ QDs as co-catalysts at the interface of light-active semiconductors increases the spectral response and reduces charge carrier recombination [18]. As a result, Ti_3_C_2_ QDs derived from 2D Ti_3_C_2_ MXene nanosheets have gained increasing attention in photocatalytic dye degradation, photocatalytic hydrogen production, and photoelectrochemical water splitting applications. The exploration of synergistic interaction between Ti_3_C_2_ QDs and various semiconductor materials is particularly advantageous for environmental remediation. Furthermore, the retained layered characteristics along with a high density of transition metal centers promote abundant active sites and increased electronic density of states for accelerating photocatalytic reactions. For instance, Ti_3_C_2_ QDs integrated with 2D perovskite Ca_2_Nb_3_O_10_ nanosheets demonstrated an improvement in photoresponse behavior [19].

**Figure 1:**
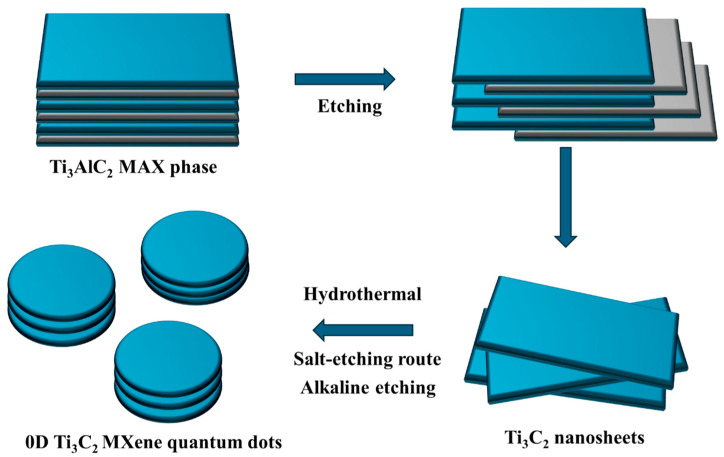
Facile growth of 0D Ti_3_C_2_ QDs from 2D Ti_3_C_2_ MXene nanosheets.

Thus, the pillaring of Ti_3_C_2_ QDs at various semiconductor interfaces has been evaluated for achieving environmental sustainability. In particular, the role of 0D Ti_3_C_2_ QDs in light-induced toxic matter degradation and hydrogen evolution has been systematically explored. The extraction of 0D Ti_3_C_2_ QDs from 2D Ti_3_C_2_ MXene nanosheets by facile hydrothermal, salt-etching, and alkaline-etching processes is illustrated in Figure 1.

## 2. Importance of Ti_3_C_2_ QDs for Photocatalytic Activity

**Figure 2:**
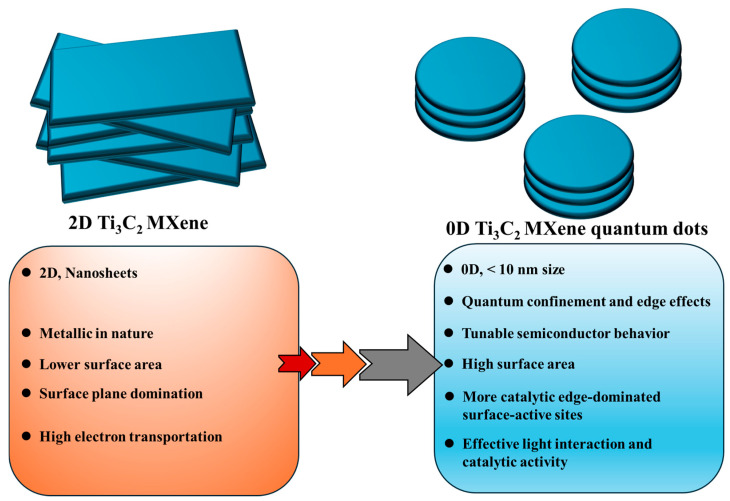
Advantages of 0D Ti_3_C_2_ QDs over 2D Ti_3_C_2_ MXene nanosheets as a potential catalyst surface during photocatalytic activity.

Generally, semiconductor materials separate the charge carriers and fulfill the photocatalytic activity by proper redox reactions [20]. Nanofibrous metal oxides, such as TiO_2_-SnO_2_, have demonstrated sustainable photocatalytic behavior [21]. But the reaction process is limited by recombination and poor diffusion of charge carriers. Ti_3_C_2_ QDs, owing to their concomitant quantum confinement and light-active semiconductor behavior, significantly enhanced the charge carrier serration efficiency [22]. Compared to metallic 2D Ti_3_C_2_ MXenes, the evolution of 0D Ti_3_C_2_ QDs smaller than 10 nm facilitates pronounced quantum confinement effects and improved light absorption characteristics. While retaining a layered structural motif similar to that of their 2D Ti_3_C_2_ MXene counterpart [23], MXene QDs additionally benefit from nanoscale effects. Furthermore, the combination of quantum confinement and metallic conductivity greatly promotes intrinsic localized surface plasmon resonance (LSPR) at the nanoscale level [24]. As a result, 0D Ti_3_C_2_ QDs with high carrier density exhibit enhanced charge carrier separation and reaction kinetics due to increased surface-active sites and surface conductivity. Such Ti_3_C_2_ QDs can adsorb more photons for facilitating efficient generation of photoelectron and holes. Moreover, 0D Ti_3_C_2_ QDs demonstrate improved corrosion stability during photocatalytic activity. The formation of a robust Schottky barrier and deeper work function at the interface of porous graphitic carbon nitride further improved the photocatalytic hydrogen evolution [25]. Therefore, 0D Ti_3_C_2_ QDs are highly promising for light-induced environmentally sustainable technologies due to their superior light absorption, charge transfer, and LSPR effects. A schematic comparison highlighting the advantages of 0D Ti_3_C_2_ QDs over 2D Ti_3_C_2_ MXene nanosheets is presented in Figure 2.

## 3. 0D Ti_3_C_2_ QD-Based Composites for Various Environmental Applications

### 3.1. Photocatalytic Toxic Matter Degradation 

Integration of 0D quantum dots and semiconductors plays a crucial role in visible light-responsive applications [26]. In this context, understanding the role of 0D Ti_3_C_2_ QDs at various semiconductor interfaces is essential for enhancing photocatalytic dye degradation under visible light irradiation. The incorporation of Ti_3_C_2_ QDs significantly increases the number of surface-active sites and pollutant adsorption capacity. Thus, the following studies highlight the potential role of Ti_3_C_2_ QDs as co-catalysts or electron transfer bridges for accelerating charge carrier separation during the photocatalytic process.

Controlling the size of QDs smaller than 10 nm has been shown to markedly enhance photocatalytic activity [27]. In relation to this, Wang et al. [28] synthesized Ti_3_C_2_ MXene QDs with a size of 8 nm and integrated them with SiC (TQDs/SiC) for NO degradation under visible light. Owing to quantum confinement effects and nanoscale dimensions, Ti_3_C_2_ MXene QDs achieved abundant unsaturated bonds on their surface and edges for promoting stronger interactions with surrounding atoms. Also, the charge transfer resistance significantly decreased at the interface through the introduction of TQDs. The specific surface area of the TQDs/SiC composite (2:10 molar ratio) increased to 81.34 m^2^/g compared to that of pure SiC (41.95 m^2^/g), which is ascribed to an increase in the surface activities of Ti_3_C_2_ MXene QDs (2–8 nm). Further, the composite exhibited a narrowed band gap of 2.08 eV with a Fermi energy level of −0.58 eV. As a result of synergistic effects, the TQDs/SiC composite achieved a NO removal efficiency of 74.6%, which was remarkably higher than that of pure SiC (24.3%) and TQDs (20.3%). Furthermore, the composite demonstrated an improvement in photostability and chemical robustness. The conduction band potential of SiC (−1.38 eV) was more negative than the Fermi energy level of TQDs (−0.39 eV) vs. NHE. In such a way, the conduction band electrons of SiC effectively transferred to TQDs, which explained the electron-accepting behavior of the TQDs. Overall, precise control over Ti_3_C_2_ MXene QDs (<10 nm) formation similar to 2D Ti_3_C_2_ MXene features was highly beneficial for stable degradation of toxic pollutants. 

**Figure 3:**
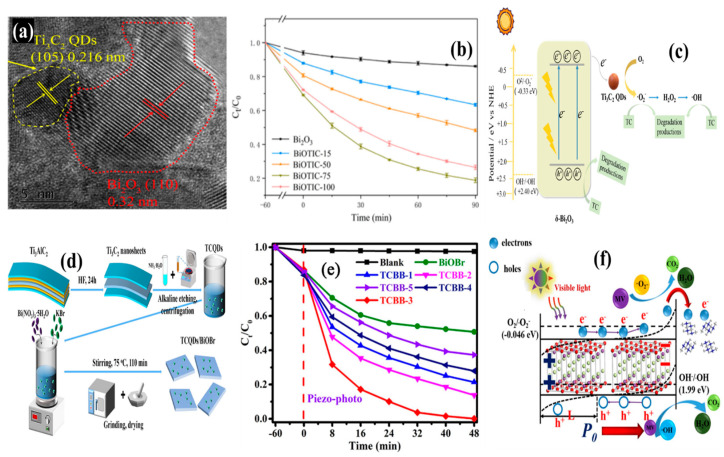
(**a**) Robust interfacial interaction between Ti_3_C_2_ QDs and Bi_2_O_3_, (**b**) tetracycline degradation ability by BiOTiC of about 82% in 90 min, (**c**) charge transportation mechanism involved in BiOTiC composite with Ti_3_C_2_ QDs as co-catalyst (reprinted from Ref. [30]; copyright with permission from Elsevier), (**d**) schematic representation of Ti_3_C_2_ QDs/BiOBr heterostructure formation, (**e**) superior methyl violet degradation of about 99.8% in 48 min by Ti_3_C_2_ QDs/BiOBr, and (**f**) charge transportation (reprinted from Ref. [32]; copyright with permission from Elsevier).

MXenes have also demonstrated strong co-catalytic behavior in photocatalytic systems [29]. Thus, the involvement of Ti_3_C_2_ MXene QDs as a co-catalyst was further investigated in a Bi_2_O_3_-based system (BiOTiC) for tetracycline degradation [30]. Here, Ti_3_C_2_ QDs with an average diameter of 3.96 nm were synthesized by a hydrothermal process (120 °C and 12 h) following successive etching (NaF+HCl, 60 °C) and exfoliation (DMSO+sonication) of Ti_3_AlC_2_ MAX and Ti_3_C_2_ MXene nanosheets. Figure 3a shows the HRTEM image, which confirms strong interfacial interaction between Ti_3_C_2_ QDs and Bi_2_O_3_. The incorporation of Ti_3_C_2_ QDs increased the surface area of the resultant BiOTiC to 39.470 m^2^/g compared to that of pure Bi_2_O_3_ (4.555 m^2^/g). Thus, the Ti_3_C_2_ QDs boosted the availability of active sites and interacted with pollutants through increased adsorption. Additionally, the light absorption range of BiOTiC extended significantly into the visible region (450 to 800 nm) compared to that of pure Bi_2_O_3_ (200 to 400 nm). The band gap was reduced to 2.71 eV (from 2.91 eV) with the incorporation of 75 mL Ti_3_C_2_ QDs. The improvements resulted in about 82% degradation of tetracycline in 90 min compared to 14% for pure Bi_2_O_3_ as shown in Figure 3b. The presence of abundant surface terminations and exposed Ti metal sites further enhanced the redox reactions. Overall, Ti_3_C_2_ QDs about 3.96 nm in size effectively enhanced the surface-active sites, light absorption, and charge carrier separation efficiencies, which led to remarkable degradation of organic compounds as shown in Figure 3c. Specifically, Ti_3_C_2_ QDs successfully accepted the electrons from the conduction band of Bi_2_O_3_.

The development of Ti_3_C_2_ MXene in the form of nanodots also offers significant advantages for toxic pollutant degradation. In this context, Lv et al. [31] uniformly grew 0D MXene nanodots on 2D Ti_3_C_2_ MXene nanosheets via a two-step salt-etching process for the degradation of multiple dyes (MB, MeB, and RhB) under sunlight. The resulting 0D/2D heterostructure interface exhibited an increased surface area of 24.8 m^2^/g compared to 8.7 m^2^/g for 2D Ti_3_C_2_ MXene. This configuration highlighted the potential role of ~7.2 nm nanodots in increasing surface area and toxic dye adsorption. Additionally, the incorporation of 0D Ti_3_C_2_ nanodots modified the band gap to 2.82 eV, improving the visible light responsiveness. Accordingly, degradation efficiencies of 84, 60, and 66% were achieved for MB, MeB, and RhB respectively. These results emphasize that controlled growth of 0D Ti_3_C_2_ MXenes significantly tailored surface chemistry for advancing the photocatalytic performance.

In another study, Yao et al. [32] increased the role of Ti_3_C_2_ QDs integrated into 2D BiOBr nanosheets for methyl violet degradation. The Ti_3_C_2_ QDs/BiOBr composite enhanced piezo-photocatalytic activity through strong interfacial coupling as shown in Figure 3d. At an optimal loading of 1% of Ti_3_C_2_ QDs to BiOBr, the surface area increased to 11.24 m^2^/g (BiOBr—9.81 m^2^/g). Interestingly, the presence of Ti_3_C_2_ QDs facilitated the generation of piezoelectric potential difference across the nanosheet system. On the other hand, the electron-accepting and accumulation behavior of Ti_3_C_2_ QDs weakened the potential. This synergistic effect significantly enhanced the piezo-photocatalysis. As a result, the integration and formation of a 0D/2D interface between Ti_3_C_2_ QDs and BiOBr nanosheets successfully degraded the methyl violet (10 mg/L) by about 99.8% in 48 min (Figure 3e). At the interface of Ti_3_C_2_, upward band bending was observed by BiOBr to prevent charge carrier recombination and its effective participation in dye degradation. Here, the high electrically conductive behavior of Ti_3_C_2_ QDs greatly attracted and accumulated charge carriers from BiOBr. Overall, mechanical stretch significantly induced a built-in piezoelectric field that accelerated the photoelectron transfer for expansion of the piezo-phototronic effect across the 0D/2D surface heterostructure (Figure 3f).

The sandwiching of Ti_3_C_2_ QDs (~5 to 7 nm) between In_2_S_3_ and SmFeO_3_ (IMS) demonstrated highly efficient photocatalytic removal of sulfamethoxazole (SMX) and 4-chlorophenol (4-CP) [33]. By the addition of 500 mg Ti_3_C_2_ MXene with 50 mL of (CH_3_)_2_NCH (stirred for 30 min and ultrasonicated for 2 h), a black mixture was achieved, which was kept in a stainless steel autoclave at 120 °C for 10 h. This procedure achieved Ti_3_C_2_ QDs of about 5 to 7 nm in size. Notably, the Ti_3_C_2_ QDs acted as an effective “charge transfer bridge” between In_2_S_3_ and SmFeO_3_ within the Z-scheme mechanism. The composite prepared with an optimal amount (3.0 mL) of Ti_3_C_2_ QDs (In_2_S_3_/3%MQDs/SmFeO_3_-IMS-3) exhibited the highest specific surface area of 80.9 m^2^/g. This optimized composite showed enhancement in redox potential and improved charge carrier separation, which was consistent with an efficient Z-scheme pathway. However, excessive loading of Ti_3_C_2_ QDs negatively affected photocatalytic activity due to (i) improved charge carrier recombination centers and (ii) blockage of active surface sites. The role of the rapid charge carrier reception and transfer process between In_2_S_3_ and SmFeO_3_ was successfully fulfilled by the introduction of Ti_3_C_2_ QDs, thus achieving efficient photocatalytic activity. Under optimal conditions, the composite achieved degradation efficiencies of 98.0 and 95.4% for SMX and 4-CP in 120 and 90 min, respectively.

**Figure 4:**
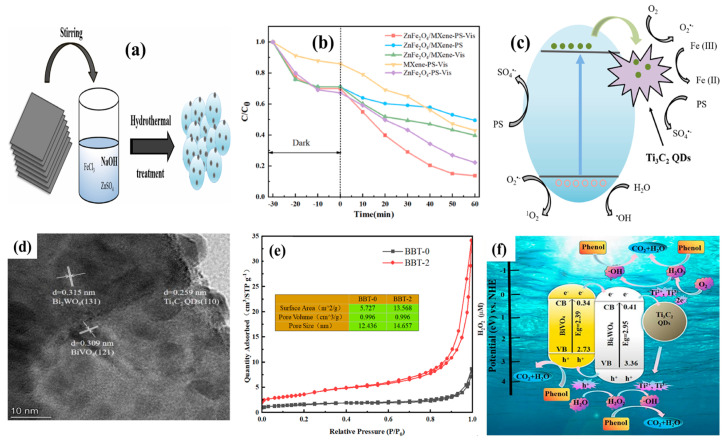
(**a**) Hydrothermal process to achieve Ti_3_C_2_QDs on ZnFe_2_O_4_, (**b**) tetracycline degradation of about 87% by the ZnFe_2_O_4_/Ti_3_C_2_ QDs-PS in the visible region in 60 min, (**c**) potential role of Ti_3_C_2_QDs for charge carrier separation with ZnFe_2_O_4_ (reprinted from Ref. [34]; copyright with permission from Elsevier), (**d**) dual heterojunction interface formation between BiVO_4_, Bi_2_WO_6_, and Ti_3_C_2_ QDs, (**e**) variation in the surface area after integration of Ti_3_C_2_ QDs, and (**f**) phenol degradation process by the BBT-2 composite in a remarkable charge transfer process (reprinted from Ref. [36]; copyright with permission from Elsevier).

Ti_3_C_2_ QDs-modified ZnFe_2_O_4_ composites exhibited rapid degradation of about 87% of the organic pollutant tetracycline within 60 min in the presence of persulfate (PS) [34]. In this system, the hydrothermal process facilitated the transformation of 2D Ti_3_C_2_ MXene nanosheets into 0D Ti_3_C_2_ QDs on the ZnFe_2_O_4_ surface as shown in Figure 4a. High-resolution analysis revealed well-defined lattice fringes of Ti_3_C_2_ QDs (0.96 nm) and ZnFe_2_O_4_ (0.25 nm), which indicated strong interfacial contact. The abundance of active edge sites and -OH termination groups on Ti_3_C_2_ QDs promoted effective adsorption of PS molecules. Furthermore, the formation of a Schottky junction between the Ti_3_C_2_ QDs and ZnFe_2_O_4_ facilitated efficient charge transfer from ZnFe_2_O_4_ to Ti_3_C_2_ QDs, which effectively suppressed charge carrier recombination and enhanced tetracycline degradation in 60 min as shown in Figure 4b. These results were higher than those of Ti_3_C_2_ MXene QDs used as a co-catalyst with Bi_2_O_3_ for photocatalytic tetracycline degradation (82% in 90 min) [30]. The potential role of Ti_3_C_2_ QDs in the presence of PS is shown in Figure 4c. Here, the Ti_3_C_2_ QDs effectively accepted the electrons from ZnFe_2_O_4_ for efficient degradation of tetracycline.

Further, the potential local surface plasmon resonance behavior of Ti_3_C_2_ QDs at the CuSe interface was investigated for photocatalytic reduction of Cr(VI) under the solar spectrum [35]. Here, Ti_3_C_2_ QDs were developed by the addition of 0.1 g Ti_3_AlC_2_ in 20 mL DI water (pH of 8) and this mixture was transferred into a 50 mL stainless steel autoclave at 100 °C for 24 h to achieve Ti_3_C_2_ QDs between 3 and 20 nm. In this system, Ti_3_C_2_ QDs functioned as an n-type material at the interface of p-type CuSe, forming a plasmonic semiconductor heterostructure. During this process, strong electrostatic interaction was established between Ti_3_C_2_ QDs and CuSe crystals, facilitating a plasmonic heterostructure. The composite thickness was maintained between 9 and 15 nm, which promoted efficient charge carrier separation and enhanced utilization of photogenerated charge carriers. By varying the Ti_3_C_2_ QD content from 10 to 60 wt%, the optimal composition (40 wt%) exhibited complete degradation of Cr(VI) in 80 min with a band gap of 1.35 eV. The conduction band potential of Ti_3_C_2_ QDs was more negative (−0.6 V) than that of CuSe (−0.41). Thus, under light irradiation, Ti_3_C_2_ QDs effectively generated hot electrons that were transferred to the conduction band of CuSe, enabling the reduction of Cr(VI) to Cr(III). The achieved plasmonic heterostructure reduced the charge transfer resistance due to the presence of Ti_3_C_2_ QDs. The existence of O-H stretching (3415 cm^−1^) and bending (1618 cm^−1^) vibrations in the resultant composite maintained the morphological stability without photocorrosion during the photocatalytic process. Overall, the generation of hot electrons in the 0D/2D MQDs/CuSe system significantly enhanced photocatalytic performance under visible light.

Further, the formation of a dual heterojunction in the BiVO_4_/Bi_2_WO_6_/Ti_3_C_2_ QDs (BBT) composite demonstrated phenol degradation via a photocatalytic Fenton process [36]. The HRTEM image of the BBT-2 composite with robust surface interaction is presented in Figure 4d. The optimized BBT-2 photocatalyst effectively suppressed charge carrier recombination, promoting charge carrier separation and its participation in photocatalytic activity through the incorporation of 5 nm Ti_3_C_2_ QDs. The involvement of Ti_3_C_2_ QDs (mass at 2%) significantly increased the surface area to 13.568 m^2^/g (BBT-2) compared to that without addition of Ti_3_C_2_ QDs (5.727 m^2^/g) (Figure 4e). Importantly, the atomic structures of the individual components remained intact during composite formation, contributing to an enhancement in the photocatalytic activity. Furthermore, the increased number of active surface sites accelerated the interaction with phenol molecules. As a result, the potential dual heterojunction system achieved approximately 78% degradation of phenol (0.5 g/L) in 180 min. The degradation efficiency remained relatively stable, decreasing from 78% to 66% over 10 cycles. Overall, Ti_3_C_2_ QDs played a crucial role in facilitating phenol degradation through effective separation of electrons from BiVO_4_ and Bi_2_WO_6_ through their interaction with H_2_O_2_ as illustrated in Figure 4f. Ti_3_C_2_ QDs greatly separated and trapped electrons from the BiVO_4_/Bi_2_WO_6_ interface through Schottky barrier formation.

The creation of oxygen vacancies in TiO_2_ and their interaction with Ti_3_C_2_ MXene can enhance the adsorption sites and modulate the electron environment [37]. In this context, oxygen-vacancy-rich W_18_O_49_ was successfully integrated with Ti_3_C_2_ QDs, resulting in improved degradation of multiple dyes [38]. Through the addition of 0.4 g of Ti_3_C_2_ MXene powder into 20 mL DI water at a pH of 9 and hydrothermal treatment at 100 °C for 6 h, Ti_3_C_2_ QDs were achieved. Oxygen vacancies were introduced into Ti_3_C_2_/W_18_O_49_ through Ar treatment at various mass ratios and durations. Notably, a composite with a 10 wt% of Ti_3_C_2_/W_18_O_49_ and 60 s of Ar treatment achieved a higher surface area of 93.9 m^2^/g than pure Ti_3_C_2_ QDs (19.6 m^2^/g). The combined effects of defect states (oxygen vacancies) and increased surface area triggered light absorption efficiency for the promotion of charge carrier generation, leading to superior photocatalytic activity. Further, -OH terminations (1636 and 3446 cm^−1^) promoted the charge transfer process in a Ti_3_C_2_/W_18_O_49_ composite. Interestingly, the electrical conductivity and quantum confinement effects of Ti_3_C_2_ QDs accelerated the photocatalytic activity. As a result, over 99% degradation efficiency was achieved for various pollutants, including 5-fluorouracil, carbamazepine, bisphenol A, and rhodamine B. In contrast, photocatalysts lacking oxygen vacancies exhibited rapid charge carrier recombination. Therefore, engineering of oxygen vacancies is crucial for attaining efficient and stable toxic matter degradation.

In another study, Cheng et al. [39] coupled Ti_3_C_2_ QDs with oxygen-vacancy-rich BiOBr hollow microspheres (TB) by a self-assembly process (4 h) for visible-light-driven degradation of tetracycline and rhodamine B. The hydrothermal process at 120 °C for 10 h resulted in Ti_3_C_2_ QDs with a size of about 5 to 8 nm, which grew on BiOBr hollow microspheres. The resulting composite displayed a higher surface area (10.96 m^2^/g) than oxygen-deficient BiOBr (7.54 m^2^/g). The incorporation of Ti_3_C_2_ QDs did not alter the morphology of BiOBr hollow microspheres, while the band gap decreased from 2.83 eV (pure BiOBr) to 2.58 eV after incorporation of Ti_3_C_2_ QDs. Additionally, the oxygen-vacancy concentration in the TB composite (28.7%) exceeded that of BiOBr (20%), which greatly influenced the photocatalytic activity. Accordingly, tetracycline degradation efficiency reached about 97.27% (BiOBr-OVs—79.07%) and 99.8% for rhodamine B (BiOBr-OVs—82%) in 120 min. The presence of -OH and -O functional groups on Ti_3_C_2_ QDs greatly triggered the charge transfer and adsorption of toxic matter for efficient photocatalytic activity. Overall, the role of oxygen vacancies effectively suppressed charge carrier recombination and facilitated electron transfer from defect states in the conduction band of BiOBr to Ti_3_C_2_ QDs.

Similarly, Qi et al. [40] anchored Ti_3_C_2_ QDs on oxygen-vacancy-rich Bi_2_WO_6_ (BWO@MQDs) by electrostatic self-assembly for the degradation of tetracycline. Specifically, Ti_3_C_2_ QDs were developed by successive DMSO (20 mL) sonication and hydrothermal processing (100 °C for 6 h) of 2D Ti_3_C_2_ MXene. The specific surface area of the resultant component significantly increased to 28.45 m^2^/g upon incorporation of Ti_3_C_2_ QDs on Bi_2_WO_6_ compared to that of pure Bi_2_WO_6_ (17.35 m^2^/g). This enhancement indicated that Ti_3_C_2_ QDs contributed to the increase in surface-active sites as well as narrowed the band gap to 2.63 eV (towards visible light) compared to that of Bi_2_WO_6_ (2.84 eV). The reduction in the band gap was mainly attributed to the altered electronic structure induced by the introduction of Ti_3_C_2_ QDs. In addition, the formation of a Schottky junction at the BWO@MQDs interface promoted the migration of photogenerated electrons from BWO to Ti_3_C_2_ QDs. In such a way, at an optimal mass ratio of 2.5% MQDs to BWO, tetracycline degradation efficiency reached 88.12% in 120 min (Bi_2_WO_6_—54.72%). Here, electrically conductive Ti_3_C_2_ QDs effectively accepted electrons from the conduction band of BWO for the degradation of tetracycline. Overall, introduction of Ti_3_C_2_ QDs effectively influenced the surface area, narrowed the band gap, and facilitated efficient charge carrier separation through the Schottky barrier under visible light irradiation.

**Figure 5:**
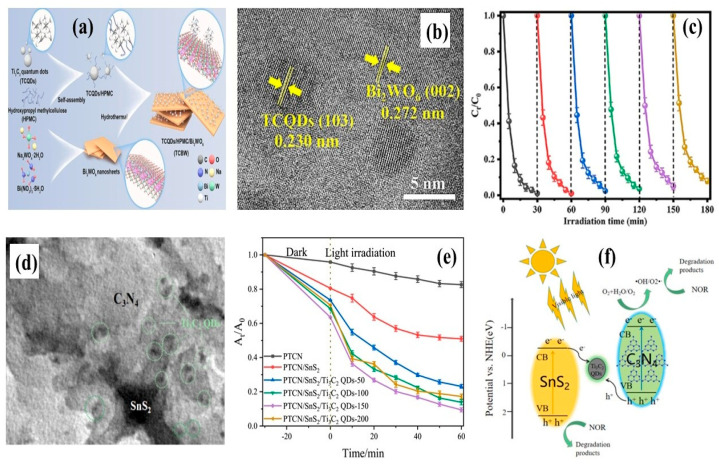
(**a**) Facile hydrothermal growth process for Ti_3_C_2_ QDs/hydroxypropyl methylcellulose/Bi_2_WO_6_ composite, (**b**) interfacial interaction between Ti_3_C_2_ QDs and Bi_2_WO_6_, (**c**) remarkable and stable tetracycline degradation ability by the TCBW composite (reprinted from Ref. [41]; copyright with permission from Elsevier), (**d**) surface morphology of Ti_3_C_2_ QDs formation in the PTCN/SnS_2_ composite, (**e**) degradation ability of norfloxacin at different contents of PTCN/SnS_2_/Ti_3_C_2_, and (**f**) Ti_3_C_2_ QDs as a bridge between PTCN and SnS_2_ for effective charge carrier separation and norfloxacin degradation (reprinted from Ref. [43]; copyright with permission from Elsevier).

In another study, Yao et al. [41] synthesized a Ti_3_C_2_ QDs/hydroxypropyl methylcellulose/Bi_2_WO_6_ (TCBW) piezo-photocatalyst through the hydrothermal method for the degradation of tetracycline under visible light, as shown in Figure 5a. The Ti_3_C_2_ QDs were uniformly distributed and strongly bonded to Bi_2_WO_6_ through hydrogen bonding, which was facilitated by the highly viscous hydroxypropyl methylcellulose containing abundant hydroxyl groups. The HRTEM image of the TCBW composite is shown in Figure 5b. The formation of a built-in electric field strengthened the piezo-phototronic effect, which promoted the efficient charge carrier transfer across the interface and thereby improved photocatalytic activity. Incorporation of Ti_3_C_2_ QDs at a mass ratio of 0.5% increased the surface area to 13.35 m^2^/g compared to that of pure Bi_2_WO_6_ (10.26 m^2^/g), while the band gap reduced from 2.75 to 2.63 eV. Accordingly, the tetracycline (20 mg/L) was degraded by about 99.6% in a very short time of 30 min, significantly outperforming pure Bi_2_WO_6_ (69%). These results were higher than those for the oxygen-vacancy-induced Bi_2_WO_6_@MQDs heterojunction (88.12%) for tetracycline degradation [40]. Overall, Ti_3_C_2_ QDs greatly enhanced pollutant degradation through strong interfacial interaction with Bi_2_WO_6_ and improved charge separation under visible light, along with excellent cyclic stability as shown in Figure 5c.

Interestingly, hydrothermal synthesis of a structurally robust 0D/2D Ti_3_C_2_ QD@Bi_2_WO_6_ composite also demonstrated effective degradation of ibuprofen under visible light [42]. Addition of about 10 mg of PEI into 2D Ti_3_C_2_ MXene, which was then processed under hydrothermal treatment (120 °C for 12 h), achieved Ti_3_C_2_ QDs. The developed Ti_3_C_2_ QDs with an average diameter of 7 nm were well dispersed on the Bi_2_WO_6_ surface. Mass ratios of 0.5% and 1% between Ti_3_C_2_ QDs and Bi_2_WO_6_ resulted in surface areas of 19.927 and 15.701 m^2^/g, respectively, providing abundant active sites for enhanced photocatalytic activity. Notably, the surface termination groups on Bi_2_WO_6_ remained unchanged even after incorporation of Ti_3_C_2_ QDs. Specifically, the structural integrity of the composite was attributed to the formation of Bi-O-Ti-C bonds, which significantly facilitated charge carrier separation via a Z-scheme mechanism from Bi_2_WO_6_ to Ti_3_C_2_ QDs. Due to negatively and positively charged Bi_2_WO_6_ and Ti_3_C_2_ QDs, downward bending of the energy band effectively transferred and accumulated the electrons on Ti_3_C_2_ QDs from Bi_2_WO_6_ for effective participation in the dye degradation process. As a result, a superior ibuprofen degradation efficiency of 97.7% was achieved at a 1% mass ratio compared to pure Bi_2_WO_6_ (80.6%) in 120 min. Overall, the transformation of 2D Ti_3_C_2_ nanosheets into 0D Ti_3_C_2_ QDs markedly improved visible light responsiveness and charge separation efficiency, which led to enhanced photocatalytic activity.

The process of tetracyanoethylene and SnS_2_ integration with Ti_3_C_2_ QDs enabled efficient degradation of norfloxacin, pefloxacin, enrofloxacin, and ciprofloxacin following the Z-scheme charge transfer mechanism [43]. The HRTEM image of the PTCN/SnS_2_/Ti_3_C_2_ composite is shown in Figure 5d. The incorporation of Ti_3_C_2_ QDs significantly increased the surface area of the resultant composite to 68.847 m^2^/g compared to that of PTCN/SnS_2_ (56.417 m^2^/g) and PTCN (10.411 m^2^/g). This phenomenon indicated that the generation of abundant reactive surface sites improved the photocatalytic activity. Additionally, the band gap decreased by 0.22 eV upon Ti_3_C_2_ QDs involvement in PTCN/SnS_2_/Ti_3_C_2_, with QDs reaching 2.38 eV compared to 2.6 eV for PTCN, which suggested improved visible light absorption. In this system, Ti_3_C_2_ QDs function as a co-catalyst facilitating charge carrier separation by capturing holes from PTCN and electrons from SnS_2_. Ti_3_C_2_ QDs with hydroxyl functional groups proved their role as an electron mediator for extending the charge transportation in the PTCN/SnS_2_/Ti_3_C_2_ composite. As a result, the degradation efficiencies for norfloxacin, pefloxacin, enrofloxacin, and ciprofloxacin were 90.53, 79.53, 81.23, and 85.62%, respectively. The degradation behavior of PTCN/SnS_2_/Ti_3_C_2_ composite toward norfloxacin is presented in Figure 5e. Moreover, this composite proved its excellent recyclability and chemical stability over five cycles. The bridging role of Ti_3_C_2_ QDs was unique in promoting effective charge separation between PTCN and SnS_2_ as shown in Figure 5f.

Very recently, Fe-MoF was interfaced with Ti_3_C_2_ QDs for visiblelight-driven degradation of methylene blue at different Ti_3_C_2_ QDs loadings (25 mg, 50 mg, and 75 mg) [44]. Addition of delaminated Ti_3_C_2_T_x_ MXene with 10 mL of TMAH (3%) treated at 120 °C for 72 h in a water bath achieved Ti_3_C_2_ QDs formation. The Ti_3_C_2_ QDs contributed to enhanced light-induced charge carrier generation and separation. At an optimal loading of 50 mg, a higher surface area of 23.463 m^2^/g and narrow band gap of 1.63 eV were achieved, which led to a photocatalytic degradation efficiency of 62.97% compared to negligible absorption in the dark state (13.21%). But pure Fe-MoF showed complete adsorption of about 73.01% without any photocatalytic activity. The above phenomenon suggests that with the addition of Ti_3_C_2_ QDs, photocatalytic activity greatly improved by suppressing the physical adsorption in the dark state. This improvement is primarily attributed to efficient charge carrier generation and transfer enabled by the visiblelight-active Ti_3_C_2_ QDs. The composite also exhibited good structural stability over three cycles without noticeable degradation due to the presence of -OH terminations on the Ti_3_C_2_ QDs. Overall, the development of robust interfacial contact between MOF and MQDs maintained an increase in toxic matter degradation.

Overall, the incorporation of 0D Ti_3_C_2_ QDs at the interface of various semiconductors effectively tuned both the surface area and band gap, enhancing the surface-active site availability and visible light responsiveness. These improvements promoted efficient charge carrier separation and utilization, leading to enhancement in the degradation of various toxic pollutants. Therefore, the transformation of 2D Ti_3_C_2_ MXene into 0D Ti_3_C_2_ QDs is crucial and necessary for advancing environmental remediation technologies. A summary of 0D Ti_3_C_2_ QDs-based composite advancements during photocatalytic dye degradation is presented in Table 1.

**Table 1:** Summary of the role of Ti_3_C_2_ QDs-interfaced semiconductors for photocatalytic degradation of various toxic dyes.

Photocatalyst (Surface Area (m^2^/g))	Target Dye	Ti_3_C_2_ QD Size (Diameter, nm)	Reaction Time (min)	Degradation Efficiency (%)		Band Gap (eV)	Stability (Cycles)	Ref.
				Base Photocatalyst	Resultant Photocatalyst			
TQDs/SiC (81.34)	NO	8	30	SiC—24.3, TQDs—20.3	74.6	1.77	4	[28]
Ti_3_C_2_QDs/Bi_2_O_3_ (39.47)	TC	2–8	90	Bi_2_O_3_—14%	82	2.71	4	[30]
0D/2D Ti_3_C_2_ (24.8)	MB, MeB, and RhB	~7.2	180	---	MB—84, MeB—60, and RhB—66	2.82	4	[31]
TCQDs/BiOBr (11.24)	Methyl violet	---	48	BiOBr-18.8	99.8	2.79	4	[32]
In_2_S_3_/MQDs/ SmFeO_3_ (80.9)	SMX, 4-CP	5–7	120, 90	SMX (In_2_S_3_—16.6, SmFeO_3_—3.8) and CP (In_2_S_3_—12.3, SmFeO_3_—4.9)	SMX—98.0, CP—95.4	---	---	[33]
Ti_3_C_2_QDs/ZnFe_2_O_4_ (---)	TC	---	60	ZnFe_2_O_4_—~76	87	---	---	[34]
Ti_3_C_2_QDs/CuSe (---)	Cr(VI)	3–20	80	CuSe—95	100	1.35	3	[35]
BiVO_4_/Bi_2_WO_6_/ Ti_3_C_2_ (13.568)	Phenol	5 nm	180	BiVO_4_/Bi_2_WO_6_—59	78.0	~2.3	10	[36]
Ti_3_C_2_/W_18_O_49_ (93.9)	5-FLU, CBZ, BPA, and RhB	---	60	---	All 100%	---	5	[38]
Ti_3_C_2_ QDs/oxygen-vacancy-rich BiOBr (10.96)	TC and RhB	5–8	120	TC (BiOBr—79.07) and (BiOBr—82)	TC—97.27 and RhB—99.8	2.58	20	[39]
Ti_3_C_2_ QDs/oxygen-vacancy Bi_2_WO_6_ (28.45)	TC	---	120	Bi_2_WO_6_—54.72	88.12	2.63	5	[40]
Ti_3_C_2_QDs/hydroxypropyl methylcellulose/Bi_2_WO_6_ (13.35)	TC	3–4	30	Bi_2_WO_6_—69	99.6	2.63	6	[41]
Ti_3_C_2_ QD@Bi_2_WO_6_ (19.927)	Ibuprofen	7	120	Bi_2_WO_6_—80.6	97.7	2.937	5	[42]
PTCN/SnS_2_/Ti_3_C_2_QD (68.847)	Norfloxacin, pefloxacin, enrofloxacin, and ciprofloxacin	---	60	---	Norfloxacin—90.53, pefloxacin—79.53, enrofloxacin—81.23, and ciprofloxacin—85.62	2.38	5	[43]
Fe-MoF/Ti_3_C_2_QD (23.463)	MB	6	4 h	Ti_3_C_2_QDs—23.33	62.97	1.63	3	[44]

### 3.2. Photocatalytic Hydrogen (H_2_) Evolution

The altered electronic structure of Ti_3_C_2_ QDs plays a crucial role in enhancing photocatalytic performance. Specifically, Ti_3_C_2_ QDs facilitate rapid charge carrier transport and act as effective electron traps for suppressing charge recombination. Thus, several studies have highlighted the significant contribution of 0D Ti_3_C_2_ QDs to photocatalytic H_2_ evolution.

**Figure 6:**
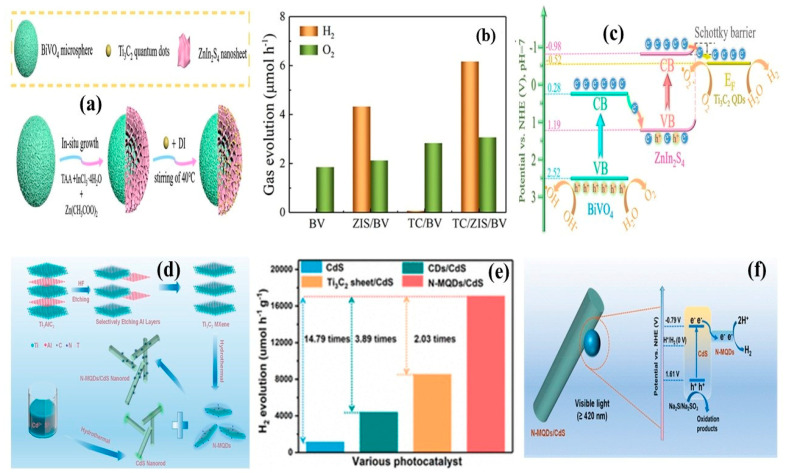
(**a**) Ti_3_C_2_ QD growth on BiVO_4_@ZnIn_2_S_4_ microspheres, (**b**) H_2_ evolution ability of 6.16 μmolh^−1^ by the BV@ZIS/TC QDs composite, (**c**) Schottky junction formation by the Ti_3_C_2_ QDs at the interface of BiVO_4_@ZnIn_2_S_4_ for efficient charge carrier separation and its participation in H_2_ production (reprinted from Ref. [45]; copyright with permission from Elsevier), (**d**) hydrothermal process for the development of the nitrogen-doped Ti_3_C_2_ QDs interface with 1D CdS nanorods, (**e**) remarkable H_2_ evolution of 17,094 μmolh^−1^g^−1^ by the N-MQDs/CdS compared to other counterparts, and (**f**) charge carrier separation ability by the Ti_3_C_2_ QDs at the interface of 1D CdS for effective H_2_ generation (reprinted from Ref. [48]; copyright with permission from ACS Publication).

The integration of Ti_3_C_2_ QDs at the interface of BiVO_4_@ZnIn_2_S_4_ (BV@ZIS) microspheres resulted in enhanced photocatalytic H_2_ evolution via a Z-scheme charge transfer mechanism [45]. A schematic representation of BiVO_4_@ZnIn_2_S_4_/Ti_3_C_2_ QDs heterostructure formation is shown in Figure 6a. A hydrothermal process at 100 °C for 6 h with the addition of 0.5 g of 2D Ti_3_C_2_ MXene in 60 mL of DI water achieved Ti_3_C_2_ QDs. The formation of a Schottky junction at the ZnIn_2_S_4_ interface induced the growth of ~10 nm Ti_3_C_2_ QDs, which effectively promoted charge carrier separation by inhibiting recombination. Specifically, Ti_3_C_2_ QDs functioned as an electron sink. Furthermore, Ti_3_C_2_ QDs extended light absorption from the visible to near-infrared region. Owing to the favorable band alignment, the BV@ZIS/TC QDs system achieved a H_2_ evolution rate of 6.16 μmolh^−1^ (BV@ZIS-4.32 μmolh^−1^) as shown in Figure 6b. Notably, the composite achieved superior light absorption at 460 nm after Ti_3_C_2_ QDs incorporation. Additionally, surface functional groups on Ti_3_C_2_ QDs provided abundant active sites for an increment in the photocatalytic activity. This composite also demonstrated efficient degradation of bisphenol of about 96.4% in 180 min. Due to the conduction band potential of ZnIn_2_S_4_ being located higher than the Fermi energy of Ti_3_C_2_ QDs and Schottky junction formation, electrically conductive Ti_3_C_2_ QDs effectively captured electrons from ZnIn_2_S_4_. The presence of Ti_3_C_2_ QDs surface functional groups tended to increase the active species, improving the photocatalytic activity. The corresponding charge separation mechanism in the BV@ZIS/TC QDs system is shown in Figure 6c.

In another study, a flower-like heterojunction composed of Ti_3_C_2_-QDs/ZnIn_2_S_4_/Ti(IV) significantly elevated photocatalytic H_2_ evolution [46]. The incorporation of Ti_3_C_2_ QDs with enriched -OH terminations increased the specific surface area of the resultant composite (117.91 m^2^/g) compared to that of pure ZnIn_2_S_4_ (98.3 m^2^/g), which provided more active sites. The Ti_3_C_2_ QDs with sizes ranging from 2 to 5 nm were uniformly distributed on the ZnIn_2_S_4_/Ti(IV) surface. The inclusion of Ti_3_C_2_ QDs enhanced light absorption towards longer wavelengths, indicating improvement in the utilization of visible light of the solar spectrum. The synergistic effect of Ti_3_C_2_ QDs and Ti(IV) co-catalysts also prolonged the charge carrier lifetime. Additionally, the band gap of the composite slightly decreased to 2.55 eV compared to that of pure ZnIn_2_S_4_ (2.59 eV). As a result, the Ti_3_C_2_-QDs/ZnIn_2_S_4_/Ti(IV) system exhibited a remarkable H_2_ evolution rate of 7.52 mmolg^−1^h^−1^, significantly outperforming that of pure ZnIn_2_S_4_ (0.47 mmolg^−1^h^−1^) and ZnIn_2_S_4_/Ti(IV) (1.87 mmolg^−1^h^−1^). The Fermi energy level of Ti_3_C_2_-QDs (−0.11 eV) was lower compared to the conduction band potential of ZnIn_2_S_4_ (−0.52 eV). Thus, Ti_3_C_2_-QDs effectively collected electrons from ZnIn_2_S_4_ through the Schottky barrier. Due to the high electrically conductive behavior of Ti_3_C_2_ QDs, electrons effectively transferred to the surface and increased H_2_ evolution. Overall, the governance of -OH-terminated Ti_3_C_2_ QDs effectively achieved stable photocatalytic H_2_ evolution under visible light irradiation.

Metal-doped TiO_2_ has been widely recognized for its effectiveness as a potential photocatalyst [47]. In this context, Ding et al. [48] successfully integrated nitrogen-doped Ti_3_C_2_ QDs with 1D CdS (N-MQDs/CdS) for enhancement of photocatalytic H_2_ evolution. Figure 6d illustrates the growth of N-MQDs on CdS nanorods by the hydrothermal process. The introduction of N-MQDs slightly increased the surface area of the N-MQDs/CdS composite to 13.24 m^2^/g compared to that of pure CdS (12.59 m^2^/g). The N-MQDs exhibited an average particle size of 3.8 nm with a thickness of 1.24 nm. Moreover, the band gap of the N-MQDs (2.25 eV) was lower relative to that of pure CdS (2.4 eV), indicating strong visible light absorption. Strong interfacial coupling between 0D and 2D nanostructures facilitated efficient electron transfer from CdS to N-MQDs. As a result, the composite achieved a remarkable photocatalytic H_2_ evolution rate of 17,094 μmolh^−1^g^−1^ at an optimal mass ratio of 3%, which was 14.79 times higher than that of pure CdS nanorods as shown in Figure 6e. Due to the stronger lower conduction band potential of CdS compared to the Fermi energy potential of N-MQDs, defective donor energy levels formed under the conduction band, which confirmed improvement in the conductivity. Thus, N-MQDs effectively collected more electrons from CdS. These findings highlighted the critical role of N-MQDs in promoting charge carrier separation and photocatalytic performance under visible light as shown in Figure 6f.

In another study, the integration of 0D Ti_3_C_2_ QDs and 2D g-C_3_N_4_ (g-C_3_N_4_@Ti_3_C_2_ QDs) significantly influenced photocatalytic activity [49]. The addition of 0.5 g of 2D Ti_3_C_2_ MXene nanosheets with 10 mL DMSO (for 24 h) processed via the hydrothermal process at 120 °C for 12 h under a nitrogen atmosphere resulted in Ti_3_C_2_ QDs. In this system, Ti_3_C_2_ QDs functioned as an efficient co-catalyst, with the ability to replace Pt and other noble metals. The well-dispersed Ti_3_C_2_ QDs on the g-C_3_N_4_ surface acted as electron acceptors, facilitating the charge transfer process from the photoexcited g-C_3_N_4_ semiconductor. The optimized composite (100 mL Ti_3_C_2_ QDs loading) exhibited a higher surface area of 40.149 m^2^/g compared to that of pure g-C_3_N_4_ (27.575 m^2^/g), which provided more surface-active sites, prolonging the charge carrier lifetime. The optical band gap of both g-C_3_N_4_ and the g-C_3_N_4_@Ti_3_C_2_ QDs composite remained about 2.56 eV. Accordingly, the g-C_3_N_4_@Ti_3_C_2_ QDs composite achieved a H_2_ evolution of 5111.8 μmolg^−1^h^−1^, which was significantly higher than that of pure g-C_3_N_4_ (196.8 μmolg^−1^h^−1^) and even Pt/g-C_3_N_4_ (1896.4 μmolg^−1^h^−1^). These results demonstrated that Ti_3_C_2_ QDs can effectively serve as a cost-effective and alternative co-catalyst to a conventional Pt co-catalyst. Overall, the incorporation of Ti_3_C_2_ QDs is highly advantageous for enhancing photocatalytic H_2_ evolution.

In another study, the incorporation of Ti_3_C_2_ QDs into an S-scheme TiO_2_/g-C_3_N_4_/Ti_3_C_2_ QDs (T-CN-TCQD) heterojunction effectively suppressed charge carrier recombination during photocatalytic H_2_ evolution [50]. The Ti_3_C_2_ QDs were uniformly anchored onto the TiO_2_/g-C_3_N_4_ surface by electrostatic interactions, forming a well-integrated morphology with a specific surface area of 36.7 m^2^/g, which was 5 times higher than that of pure TiO_2_. This enhanced surface area facilitated greater interaction with water molecules, thereby improving H_2_ evolution efficiency. Additionally, the band gap of T-CN-TCQDs (2.45 eV) was narrower than that of T-CN (2.57 eV), CN (3.22 eV), and T (2.86 eV), highlighting the significant role of Ti_3_C_2_ QD involvement. The introduction of Ti_3_C_2_ QDs promoted effective photogenerated electron separation with electrons preferentially accumulating in the electrically conductive Ti_3_C_2_ QDs. As a result, T-CN-TCQDs exhibited a H_2_ evolution rate of 5540.21 μmolh^−1^g^−1^ at a selective mass fraction of 4 wt%, along with remarkable cyclic stability over four cycles. In comparison, TiO_2_ and TiO_2_/g-C_3_N_4_ demonstrated much lower H_2_ evolution rates of 56.22 and 2847.57 μmolh^−1^g^−1^, respectively. Due to the presence of abundant functional groups (-O, -F and -OH) on TCQDs, the charge density was enriched in TCQDs compared to that in g-C_3_N_4_, enhancing the H_2_ production. Overall, the TCQDs play a crucial role in enhancing charge separation and improving photocatalytic H_2_ evolution by placing the Fermi energy level under that of CN.

**Figure 7:**
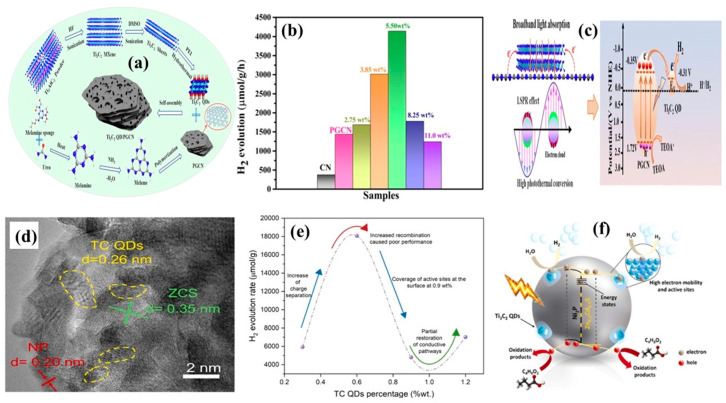
(**a**) The formation of the Ti_3_C_2_ QDs/PGCN composite, (**b**) remarkable H_2_ evolution of 4040.95 μmolg^−1^h^−1^ at 5.5 wt% through Ti_3_C_2_ QDs, (**c**) LSPR behavior by the Ti_3_C_2_ QDs for broadband light absorption and charge carrier separation for H_2_ generation at the PGCN interface (reprinted from Ref. [25]; copyright with permission from Elsevier), (**d**) the perfect interfacial interaction between NP, ZCS, and TC QDs, (**e**) variation in the H_2_ evolution at different contents of Ti_3_C_2_ QDs, and (**f**) charge carrier separation ability by the NP/ZCS@TC QDs composite for H_2_ generation (reprinted from Ref. [52]; copyright with permission from Elsevier).

In another study, Li et al. [25] investigated the LSPR behavior of Ti_3_C_2_ QDs at the interface of porous graphitic carbon nitride (Ti_3_C_2_ QDs/PGCN). Previously, the LSPR effect of Ti_3_C_2_ QDs at the CuSe interface was reported for photocatalytic degradation of Cr(VI) [35]. The schematic representation of Ti_3_C_2_ QDs/PGCN composite growth is shown in Figure 7a. Here, incorporation of Ti_3_C_2_ QDs did not significantly alter the surface area of the Ti_3_C_2_ QDs/PGCN composite, which remained at 56 m^2^/g due to the presence of abundant -OH surface termination groups. Moreover, Ti_3_C_2_ QDs preserved the structural integrity of PGCN during heterojunction formation while establishing strong interfacial contact, enabling efficient charge transfer from PGCN to Ti_3_C_2_ QDs. Specifically, the effects of a broadened light absorption range and enhanced charge carrier separation boosted the photocatalytic activity. Through the combined effects of the co-catalyst and LSPR, the Ti_3_C_2_ QDs/PGCN system achieved a H_2_ evolution of 4040.95 μmolg^−1^h^−1^ at 5.5 wt% of Ti_3_C_2_ QDs, which was significantly higher than that of PGCN (1144.74 μmolg^−1^h^−1^) as shown in Figure 7b. The Fermi energy level of Ti_3_C_2_ QDs was located at −0.31 V compared to the conduction band potential of PGCN (−0.35 V), which greatly enhanced the charge carrier separation through the involvement of Ti_3_C_2_ QDs. Overall, the introduction of Ti_3_C_2_ QDs substantially benefited photocatalytic H_2_ evolution. The LSPR behavior of Ti_3_C_2_ QDs for charge carrier separation in the Ti_3_C_2_ QDs/PGCN composite is shown in Figure 7c.

The construction of cobalt phosphite (CoP) and a Ti_3_C_2_ QDs co-modified g-C_3_N_4_ n-type heterojunction (CoP/g-C_3_N_4_/Ti_3_C_2_ QDs) significantly enhanced the photocatalytic H_2_ evolution [51]. The incorporation of Ti_3_C_2_ QDs effectively mitigated charge carrier recombination and improved charge carrier separation and transportation within the semiconducting g-C_3_N_4_ matrix. The composite exhibited a specific surface area of 42.72 m^2^/g, which was higher than that of pure g-C_3_N_4_ (30.33 m^2^/g), thereby improving surface accessibility and facilitating efficient charge transport. The band gap of CoP/g-C_3_N_4_/Ti_3_C_2_ was determined to be 2.98 eV, enabling effective incident light absorption. It should be noted that Ti_3_C_2_ QDs functioned as electron acceptors, enhancing charge carrier separation and suppressing recombination. In such a way, this composite achieved a photocatalytic H_2_ evolution of 966 μmolg^−1^h^−1^, which was significantly higher than that of pure g-C_3_N_4_ (~150 μmolg^−1^h^−1^). Overall, the involvement of Ti_3_C_2_ QDs is highly beneficial for improvement in photocatalytic H_2_ evolution.

Very recently, Hamdan et al. [52] engineered a Ti_3_C_2_ QDs-modified 2D/3D Ni_2_P/Zn_0.5_Cd_0.5_S composite (NP/ZCS@TC QDs) with Ti_3_C_2_ QDs sizes ranging from 2 to 4 nm for enhancement of photocatalytic H_2_ evolution. This composite demonstrated a good structural integrity with a surface area of 17.06 m^2^/g, exceeding that of Ni_2_P (14.19 m^2^/g) and Zn_0.5_Cd_0.5_S (15.65 m^2^/g). The HRTEM image confirmed strong interfacial interactions among NP, ZCS, and Ti_3_C_2_ QDs as shown in Figure 7d. The presence of surface functional groups, particularly hydroxyl groups (-OH), improved the stability of Ti_3_C_2_ QDs in aqueous environments. Furthermore, Ti_3_C_2_ QDs enhanced the optical absorption, interfacial charge transfer, and charge carrier lifetimes due to high electrical conductivity and zero-dimensional characteristics. Surface chemical modification induced by Ti_3_C_2_ QDs preserved the structural integrity in the composite while introducing abundant active sites through surface oxidation and functionalized metal centers. The NP/ZCS@TC QDs greatly reduced the charge transfer resistance to 0.124 kΩ, which suggests the role of electrically conductive Ti_3_C_2_ QDs. The surface oxidation and functionalized metal sites of Ti_3_C_2_ QDs provided superior surface-active sites and maintained chemical stability in the resultant photocatalyst. However, both insufficient and excessive loading of Ti_3_C_2_ QDs adversely affected photocatalytic performance. In detail, low loading limited conductive pathways, whereas high loading caused light shielding and reduced photocurrent. At an optimal loading of 0.6 wt%, Ti_3_C_2_ QDs achieved a remarkable H_2_ evolution of 18.07 mmol g^−1^_cat_, which was 21 times higher than that of ZCS as illustrated in Figure 7e. Overall, the introduction of Ti_3_C_2_ QDs significantly enhanced surface-active chemical sites, improving the photocatalytic H_2_ evolution. The H_2_ evolution performance by the potential NP/ZCS@TC QDs composite is shown in Figure 7f.

Similar to the previously reported piezo-photocatalytic process in Ti_3_C_2_ QDs/BiOBr [32] and Ti_3_C_2_ QDs/hydroxypropyl methylcellulose/Bi_2_WO_6_ [41], Wang et al. [53] investigated piezo-photocatalytic H_2_ production using 0D Ti_3_C_2_ QDs-modified 1D BaTiO_3_ nanowires. At an optimal weight ratio of 10:90 (Ti_3_C_2_ QDs to BaTiO_3_), the composite achieved a high surface area of 78.7 m^2^/g and a band gap of 3.13 eV. The incorporation of Ti_3_C_2_ QDs extended light absorption capacity and suppressed the charge recombination. Specifically, maintaining selective loading of Ti_3_C_2_ QDs enhanced the photocurrent response. Excessive loading led to Ti_3_C_2_ QDs agglomeration, reducing interfacial interactions. Under optimal conditions, the photocatalytic H_2_ evolution rate reached 481.2 μmolg^−1^h^−1^, which was 3.81 times higher than that of pure BaTiO_3_ (126.2 μmolg^−1^h^−1^). Notably, under piezo-photocatalytic conditions, an even higher H_2_ production of 1315.4 μmolg^−1^h^−1^ was achieved compared to that of BaTiO_3_ (305.6 μmolg^−1^h^−1^). These results demonstrate that the 0D/1D Ti_3_C_2_ QDs/BaTiO_3_ interface significantly enhanced both photocatalytic and piezo-photocatalytic performances.

Overall, the involvement of 0D Ti_3_C_2_ QDs as a co-catalyst at the nm size effectively modulated the surface chemical states of resultant composite systems, leading to improvements in the charge carrier separation and enhanced H_2_ evolution. Notably, Ti_3_C_2_ QDs show strong potential as alternatives to Pt-based co-catalysts due to their excellent charge separation capability across various semiconductor systems. The improvements in 0D Ti_3_C_2_ QDs-based composites for photocatalytic H_2_ production are tabulated in Table 2.

**Table 2:** Photocatalytic H_2_ generation by Ti_3_C_2_ QD-interfaced semiconductors.

Photocatalyst (Amount)	BET Surface Area (m^2^g^−1^)	Ti_3_C_2_ QDs Size (Diameter (nm))	H_2_ Evolution		Cyclic Stability (Cycles)	Apparent Quantum Yield (%)	Band Gap (eV)	Ref.
			Base Catalyst	Resultant Composite				
BiVO_4_@ZnIn_2_S_4_/ Ti_3_C_2_QDs (60 mg)	---	~10	BV@ZIS—4.32 μmolh^−1^	6.16 μmolh^−1^	4	2.9 at 460 nm	---	[45]
Ti_3_C_2_QDs/ZnIn_2_S_4_/Ti(IV) (20 mg)	117.91	2–5	ZnIn_2_S_4_—0.47 mmolg^−1^h^−1^	7.52 mmolg^−1^h^−1^	4	6.22 at 420 nm	2.55	[46]
N-Ti_3_C_2_QDs/CdS (20 mg)	13.24	3.8	CdS—1155 μmolh^−1^g^−1^	17,094 μmolh^−1^g^−1^	4	---	2.25	[48]
g-C_3_N_4_@Ti_3_C_2_ QDs (10 mg)	40.149	---	g-C_3_N_4_—196.8 μmolg^−1^h^−1^	5111.8 μmolg^−1^h^−1^	3	3.654	2.56	[49]
TiO_2_/g-C_3_N_4_/Ti_3_C_2_ QDs (50 mg)	36.7	---	TiO_2_/g-C_3_N_4_—2847.57 μmolh^−1^g^−1^	5540.21 μmolh^−1^g^−1^	4	5.81 at 420 nm	2.45	[50]
Ti_3_C_2_ QDs/PGCN	56	~1.65	PGCN—1144.74 μmolg^−1^h^−1^	4040.95 μmolg^−1^h^−1^	3	11.8 at 420 nm	---	[25]
CoP/g-C_3_N_4_/Ti_3_C_2_ QDs (10 mg)	42.72	---	g-C_3_N_4_—~150 μmolg^−1^h^−1^	966 μmolg^−1^h^−1^	5	7.6 at 420 nm	2.98	[51]
Ni_2_P/Zn_0.5_Cd_0.5_/Ti_3_C_2_ QDs	17.06	2–4	---	3011 mmol g^−1^_cat_h^−1^	---	---	2.4	[52]
Ti_3_C_2_ QDs/BaTiO_3_	78.7	---	BaTiO_3_—126.2 μmolg^−1^h^−1^	481.2 μmolg^−1^h^−1^	---	---	3.13	[53]

### 3.3. Photoelectrochemical Water Splitting

In addition to the aforementioned advancements in photocatalytic dye degradation and H_2_ evolution enabled by the evolution of Ti_3_C_2_ QDs, photoelectrochemical (PEC) water splitting has also gained significant attention through the development of composite photoelectrodes on ITO or FTO substrates. The following studies highlight the role of Ti_3_C_2_ QDs in enhancing PEC water splitting performance.

Tang et al. [54] developed a Janus-structured cobalt nanoparticle-coupled Ti_3_C_2_ QDs photoanode (Co-MQD) on FTO substrates, which facilitated the formation of a Schottky barrier for efficient charge carrier separation. The Ti_3_C_2_ QDs exhibited sizes below 10 nm with band gaps of 2.16 and 1.96 eV for Ti_3_C_2_ QDs and Co-MQDs, respectively. The surface plasmonic effects of the Co-MQDs significantly enhanced the visible light absorption, generating a large number of charge carriers for PEC activity. Plasmon-induced hot electrons from Co were injected into the Ti_3_C_2_ QDs. Later, the holes in Ti_3_C_2_ QDs transferred to the Co ground state. This phenomenon resulted in efficient charge carrier separation with a separation efficiency of 95.6% and its participation in photocatalytic activity. This process contributed to an enhancement in H_2_ evolution at the Pt electrode. As a result, a superior photocurrent density of 2.99 mA/cm^2^ was achieved at an optimal Co/Ti ratio of 48%. These results demonstrated that the Co-MQD hybrid structure provides improvement in visible light response and efficient charge carrier separation.

**Figure 8:**
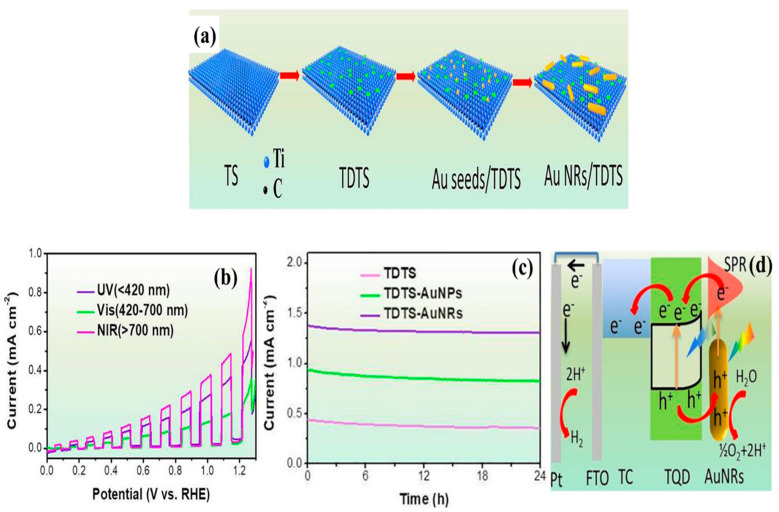
(**a**) Schematic representation of Ti_3_C_2_ QDs (dispersed on Ti_3_C_2_ nanosheets)–TDTS interaction with gold nanorod formation, (**b**) variation in the photocurrent density of the developed composite in UV, visible, NIR regions, (**c**) stable photocurrent density of the TDTS-AuNRs under white light, and (**d**) charge carrier generation and separation process in the resultant composite for PEC water splitting on FTO substrate (reprinted from Ref. [55]; copyright with permission from Elsevier).

In another study, gold nanorods were uniformly dispersed on Ti_3_C_2_ QDs, which anchored on Ti_3_C_2_ nanosheets (TDTS) to enable full-spectrum solar-driven PEC water splitting [55]. The Ti_3_C_2_ QDs, with sizes ranging from 1 to 6.0 nm, were well distributed across the nanosheets. The growth of Ti_3_C_2_ QDs dispersed on Ti_3_C_2_ nanosheets interfaced with gold nanoparticles is presented in Figure 8a. The interface between Ti_3_C_2_ QDs and Au nanorods formed a Schottky junction, promoted the transfer of plasmon-induced hot electrons from Au to the Ti_3_C_2_ QDs. On the other hand, the holes generated in Ti_3_C_2_ QDs migrated to the Au nanorods. This phenomenon triggered the suppression of charge carrier recombination and its effective participation in PEC water splitting. Moreover, Ti_3_C_2_ QDs paved a potential path for absorption of UV light. In such a way, the Au NRs/TDTS system achieved photocurrent densities of 0.48, 0.23, and 0.68 mA/cm^2^ under UV, visible, and NIR irradiation, respectively, as shown in Figure 8b. The composite also exhibited a stable photocurrent density under white light as shown in Figure 8c. Furthermore, the H_2_ evolution rate reached 12.76 μmolh^−1^ through the Au NRs/TDTS, which was significantly higher than that of Au NRs/Ti_3_C_2_ nanosheets (7.31 μmolh^−1^) under simulated solar light. Thus, these findings confirm that introduction of Ti_3_C_2_ QDs effectively suppressed charge carrier recombination for effective PEC water splitting as shown in Figure 8d.

Furthermore, the integration of Ti_3_C_2_ MXene QDs (~5 nm) and MoO_x_ nanoparticles with a BiVO_4_ porous array significantly improved the PEC water splitting efficiency [56]. The enhanced performance was attributed to rapid charge transport and improved interfacial charge separation facilitated by the MoO_x_/MQDs within the BiVO_4_ and NiFeOOH. The optimized composite achieved a photocurrent density of 5.85 mA cm^−2^ at 1.23 V vs. RHE, which was substantially higher than that of pure BiVO_4_ (1.51 mA cm^−2^) in 0.5 M KBi electrolyte. The involvement of Ti_3_C_2_ QDs resulted in a higher charge carrier density of 1.85 × 10^20^ cm^−3^ and ABPE of 2.43% at 0.58 V vs. RHE (BiVO_4_—0.46%). Notably, the Ti_3_C_2_ QDs-integrated composite maintained 97.5% photocurrent generation stability after 100 h. Overall, incorporation of Ti_3_C_2_ QDs with BiVO_4_ and MoO_x_ significantly strengthened the PEC reaction kinetics and charge carrier separation efficiency.

**Figure 9:**
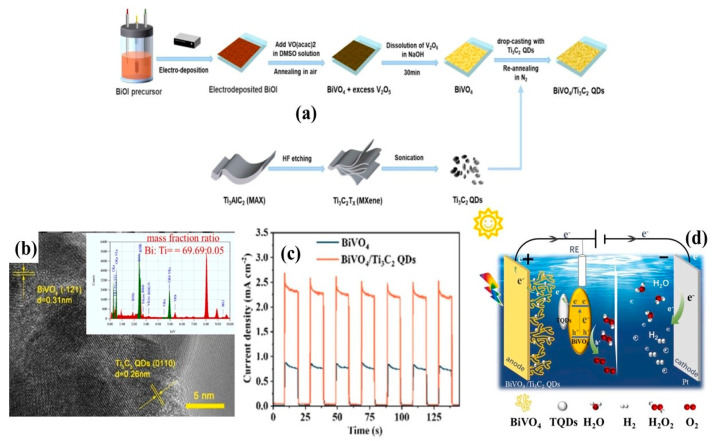
(**a**) Formation of BiVO_4_ and Ti_3_C_2_ QDs on FTO substrate, (**b**) HRTEM image of BiVO_4_ and Ti_3_C_2_ QDs interface formation, (**c**) remarkable photocurrent improvement of about 2.44 mA/cm^2^ in the BiVO_4_/Ti_3_C_2_ QDs interface at 1.23 V vs. RHE, and (**d**) PEC water splitting by the BiVO_4_/Ti_3_C_2_ QD composite on FTO substrate (reprinted from Ref. [57]; copyright with permission from Elsevier).

Recently, Li et al. [57] successfully integrated BiVO_4_ with Ti_3_C_2_ QDs on FTO substrate by a two-step calcination process to enhance the PEC water splitting activity (Figure 9a). The incorporation of Ti_3_C_2_ QDs did not alter the band gap of the composite, which remained at 2.47 eV. A robust interfacial interaction between BiVO_4_ and Ti_3_C_2_ QDs was confirmed as shown in Figure 9b. Compared to pure BiVO_4_, the Ti_3_C_2_ QDs-modified composite exhibited a higher photocurrent density. The uniform dispersion of Ti_3_C_2_ QDs on BiVO_4_ acted as a protective layer, suppressing the photoanodic dissolution of V^5+^. In addition, the formation of a Schottky barrier at the BiVO_4_/Ti_3_C_2_ QDs interface facilitated efficient charge carrier separation. As a result, the composite achieved a photocurrent density of 2.44 mA/cm^2^ at 1.23 V vs. RHE, which was markedly higher than that of pure BiVO_4_ (0.75 mA/cm^2^) (Figure 9c). Interestingly, a linear increment in the LSV curves with respect to applied bias potential was observed, which further indicates the enhancement in the charge separation efficiency. Moreover, the Ti_3_C_2_ QDs-modified electrode maintained stable photocurrent generation over 3600 s. Thus, the involvement of Ti_3_C_2_ QDs significantly improved PEC water splitting performance by promoting charge carrier separation and stability as shown in Figure 9d.

Thus, the development of Ti_3_C_2_ QDs-based photoelectrodes on ITO or FTO substrates represents an effective strategy for achieving stable PEC water splitting, as evidenced by the linear improvement in photocurrent with applied bias voltage. The role of 0D Ti_3_C_2_ QDs-based composites for photoelectrochemical water splitting is presented in Table 3.

## 4. Chemical Stability of Ti_3_C_2_ QDs During Photocatalytic Activity

**Figure 10:**
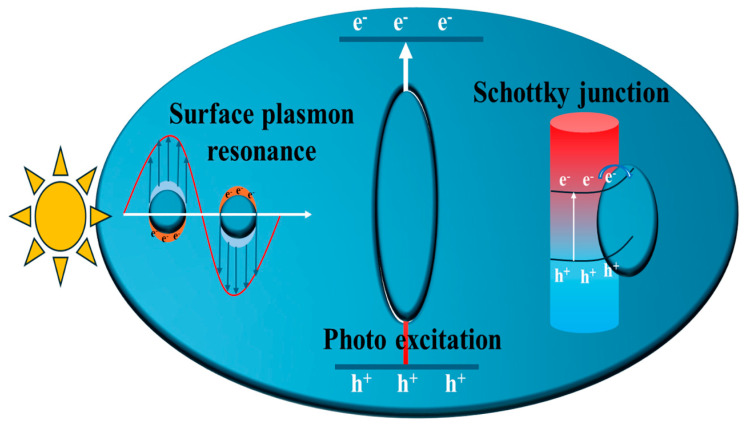
Key features involved in various light-active Ti_3_C_2_ QD-interfaced semiconductors for efficient charge carrier separation and transportation.

The development and integration of Ti_3_C_2_ QDs with various semiconductors has been strongly driven by their excellent chemical and photostability with negligible corrosion [28]. The presence of advanced surface termination groups and abundant surface-active sites further enhanced the stability of Ti_3_C_2_ QDs in an aqueous environment [52]. Photoelectrodes incorporating Ti_3_C_2_ QDs, such as Co-Ti_3_C_2_ QDs [54] and BiVO_4_/MoO_x_/Ti_3_C_2_ [56], developed on FTO substrate demonstrated remarkable stability of up to 10 h and 100 h in 1M NaOH and 0.5 M KBi electrolytes, respectively. These results highlight the excellent durability of the developed photoelectrodes under electrolyte conditions. Furthermore, the Ti_3_C_2_ QDs exhibited excellent dispersion in water, ensuring strong colloidal stability and sustained chemical interactions that enhance photocatalytic performance. Thus, Ti_3_C_2_ QDs play a crucial role in advancing the photocatalytic processes for achieving environmental sustainability. Figure 10 illustrates the key features of Ti_3_C_2_ QDs, including surface plasmon resonance, enhanced light response, and Schottky barrier formation for efficient charge carrier separation and transportation.

## 5. Conclusions

In summary, the evolution of electrochemically active 0D Ti_3_C_2_ QDs has significantly advanced photocatalytic applications at various semiconductor interfaces. Precise control over Ti_3_C_2_ QDs size and surface functional groups critically influenced the hybridization with various semiconductors and photocatalytic efficiency. Specifically, incorporation of Ti_3_C_2_ QDs increased the resultant surface area and availability of active sites in metal oxide nanostructures, enabling improved interaction with electrolytes and enhanced corrosion resistance. Understanding key properties such as band gap tuning, plasmonic behavior, and Schottky barrier formation made Ti_3_C_2_ QDs/semiconductor potential interfaces for superior charge separation at reduced recombination losses. Optimizing the Ti_3_C_2_ QDs MXene content (without agglomeration) ensures strong interfacial bonding and effective surface modification between the QDs and semiconductor materials. This behavior inevitably leads to improvement in the photocatalyst stability and enhanced redox activity over extended periods. Overall, the development of 0D Ti_3_C_2_ QDs holds significant promise for sustainable environmental applications, particularly in photocatalytic degradation of toxic pollutants and clean H_2_ evolution.

## 6. Challenges and Future Perspectives

The key transformation of 2D Ti_3_C_2_ MXene into 0D Ti_3_C_2_ QDs has gained considerable attention due to its potential involvement in photocatalytic applications. Specifically, the high surface area of Ti_3_C_2_ QDs plays a vital role in pollutant adsorption and H_2_ generation. However, there are some remaining challenges that need to be overcome to improve the photocatalytic activity of 0D Ti_3_C_2_ QDs-based semiconductors.

1. Precise size control of Ti_3_C_2_ QDs below 10 nm still remains challenging, which may produce defect-induced functional groups that can hinder photocatalytic performance. Additionally, Ti_3_C_2_ QDs aggregation may reduce stability during reactions.
2. Tailoring of selective surface termination groups is challenging due to the high surface-to-volume ratio, limiting control over electrical, optical, and chemical properties.
3. Compared to 2D Ti_3_C_2_ nanosheets, Ti_3_C_2_ QDs exhibit lower electrical conductivity. But 0D Ti_3_C_2_ MXenes are very useful with a high surface area and band gap through active quantum confinement and edge effects.
4. The band gap of pure Ti_3_C_2_ QDs is not consistently defined, and its light absorption capacity remains suboptimal. Further studies are needed to optimize these properties in heterostructures.
5. Achieving optimal band alignment and strong interfacial interactions with various semiconductors is essential to enhance charge separation and reduce charge transfer resistance.
6. Incorporating single-atom catalysts or doping Ti_3_C_2_ QDs with single atoms can increase the number of active sites for further improvement in photocatalytic activity.
7. Modifying the Ti_3_C_2_ QDs with amino groups during hydrothermal treatment further enhances the electron-accepting behavior and suppresses recombination during photocatalytic activity.

In conclusion, advancing the synthesis and engineering of 0D Ti_3_C_2_ QDs is essential for unlocking new charge separation pathways. This strategy enhances photocatalytic efficiency for pollutant degradation and H_2_ evolution, contributing to a sustainable environment.

## Figures and Tables

**Table 3:** Photoelectrochemical water splitting performance of various Ti_3_C_2_ QDs-interfaced semiconductors.

Photocatalyst (Substrate)	Electrolyte/ Light Intensity	Potential vs. Reference Electrode	Band Gap (eV)	Charge Carrier Density/ Separation/ Injection (%)	IPCE/ABPE (%)	Photocurrent Density		Stability	Ref.
						Base Material	Resultant Composite		
*Ti_3_C_2_ MXene QDs for photoelectrochemical water splitting*									
Co-Ti_3_C_2_QDs (FTO)	1 M NaOH/ 1 Sun	vs. RHE	1.96	---/95.6/---	---/0.93% at 0.44 V	Ti_3_C_2_QDs-1.54 mA/cm^2^	2.99 mA/cm^2^	10 h	[54]
Au NRs/Ti_3_C_2_ nanosheets/Ti_3_C_2_ QDs (FTO)	1 M KOH/ 1 Sun	vs. RHE	---	(4.37 × 10^21^ cm^−3^)/---/---	8% at 300 nm/---	---	0.48 (UV), 0.23 (Visible), and 0.68 (NIR) mA/cm^2^	---	[55]
BiVO_4_/MoO_x_/ Ti_3_C_2_ (FTO)	0.5 M KBi/1 Sun	vs. RHE	---	---/91/---	90% at 380–440 nm/2.43% at 0.58 V	BiVO_4_—1.51 mA cm^−2^	5.85 mA/cm^2^	100 h	[56]
BiVO_4_/ Ti_3_C_2_ QDs (FTO)	0.5 M NaSO_4_/ 1 Sun	vs. RHE	2.47	---/---/100	---/0.2965% at 0.94 V	BiVO_4_—2.99 mA/cm^2^	2.44 mA/cm^2^	3600 s	[57]

## Data Availability

Data will be made available on request.

## References

[1] Naguib M., Kurtoglu M., Presser V., Lu J., Niu J., Heon M., Hultman L., Gogotsi Y., Barsoum M.W. (2011). Two-dimensional nanocrystals produced by exfoliation of Ti_3_AlC_2_. Adv. Mater..

[2] Sreedhar A., Noh J.-S. (2021). Recent advances in partially and completely derived 2D Ti_3_C_2_ MXene based TiO_2_ nanocomposites towards photocatalytic applications: A review. Sol. Energy.

[3] Sreedhar A., Pallavolu M.R., Noh J.S. (2023). Progress in surface and interlayer distance modulated 2D Ti_3_C_2_ MXenes for potential flexible supercapacitors: A review. Appl. Mater. Today.

[4] Sreedhar A., Noh J.-S. (2025). Role of emerging 2D Nb_2_CT_x_ MXene for multiple toxic matter degradation, H_2_ generation, and H_2_ storage applications: A review. J. Water Process Eng..

[5] Gogotsi Y., Huang Q. (2021). MXenes: Two-dimensional building blocks for future materials and devices. ACS Nano.

[6] Chandrasekar N., Alexander P.S., Pham P.T., Ramachandran B., Nivedha D.R., Shekar N., Muraleetharan M., Kiran S.K., Hwang M.T. (2025). Exploring the potential of MXenes for biomedical and environmental applications—An abridged review. Luminescence.

[7] Misra M., Sreedhar A., Noh J.-S. (2025). Advancements in 2D Ti_3_C_2_ MXene interfaced various metal oxide semiconductors for photoelectrochemical water splitting: A review. Microchem. J..

[8] Guan C., Yue X., Fan J., Xiang Q. (2022). MXene quantum dots of Ti_3_C_2_: Properties, synthesis, and energy-related applications. Chin. J. Catal..

[9] Matyszczak G., Yedzikhanau A., Jasiak C., Bojko N., Krawczyk K. (2025). Applications of quantum dots in photo-based advanced oxidation processes for the degradation of contaminants of emerging concern—A Review. Catalysts.

[10] Xue Q., Zhang H., Zhu M., Pei Z., Li H., Wang Z., Huang Y., Huang Y., Deng Q., Zhou J. (2017). Photoluminescent Ti_3_C_2_ MXene quantum dots for multicolor cellular imaging. Adv. Mater..

[11] Shao B., Liu Z., Zeng G., Wang H., Liang Q., He Q., Cheng M., Zhou C., Jiang L., Song B. (2020). Two-dimensional transition metal carbide and nitride (MXene) derived quantum dots (QDs): Synthesis, properties, applications and prospects. J. Mater. Chem. A.

[12] Mubarak N.M. (2026). Sustainable development of MXene quantum dots-based material for energy harvesting. J. Environ. Chem. Eng..

[13] Mimona M.A., Rimon M.I.H., Zohura F.T., Sony J.M., Rim S.I., Arup M.M.R., Mobarak M.H. (2025). Quantum dot nanomaterials: Empowering advances in optoelectronic devices. Chem. Eng. J. Adv..

[14] Singh A., Mahapatra S., Prasad R., Singh S.K., Chandra P. (2025). Optoelectronic MXene quantum dots: Frontiers in sensor technology for next-generation diagnostics and environmental monitoring. Nanoscale.

[15] Cheng Y., Jiang B., Chaemchuen S., Verpoort F., Kou Z. (2023). Advances and challenges in designing MXene quantum dots for sensors. Carbon Neutralization.

[16] Yang F., Ge Y., Yin T., Guo J., Zhang F., Tang X., Qiu M., Liang W., Xu N., Wang C. (2020). Ti_3_C_2_T_x_ MXene _Q_uantum _D_ots with enhanced stability for ultrafast photonics. ACS Appl. Nano Mater..

[17] Hu B., Chen J., Gao Z., Chen L., Cao T., Li H., Yu Q., Wang C., Gan Z. (2024). Biodegradable MXene quantum dots with high near-infrared photothermal performance for cancer treatment. ACS Appl. Bio Mater..

[18] Zhu Y., Feng L., Zhao R., Liu B., Yang P. (2024). Review of MXene-derived quantum dots for cancer theranostics. ACS Appl. Nano Mater..

[19] Zheng Y., Wang Y., Li Z., Yuan Z., Guo S., Lou Z., Han W., Shen G., Wang L. (2023). MXene quantum dots/perovskite heterostructure enabling highly specific ultraviolet detection for skin prevention. Matter.

[20] Ly N.H., Gnanasekaran L., Aminabhavi T.M., Vasseghian Y., Joo S.-W. (2025). Photogenerated charge carriers in photocatalytic materials for solar hydrogen evolution. Curr. Opin. Chem. Eng..

[21] Babu V.J., Rashid S.S.A.A.H., Sundarrajan S., Ramakrishna S. (2023). Metal oxide electrospun nanofibrous membranes for effective dye degradation and sustainable photocatalysis. Sustain. Chem..

[22] Bimberg D., Pohl U.W. (2011). Quantum dots: Promises and accomplishments. Mater. Today.

[23] Mohanty B., Giri L., Jena B.K. (2021). MXene-derived quantum dots for energy conversion and storage applications. Energy Fuels.

[24] Darwish M.A., Abd-Elaziem W., Elsheikh A., Zayed A.A. (2024). Advancements in nanomaterials for nanosensors: A comprehensive review. Nanoscale Adv..

[25] Li J., Peng H., Luo B., Cao J., Ma L., Jing D. (2023). The enhanced photocatalytic and photothermal effects of Ti_3_C_2_ Mxene quantum dot/macroscopic porous graphitic carbon nitride heterojunction for Hydrogen Production. J. Colloid Interface Sci..

[26] Gong S., Teng X., Niu Y., Liu X., Xu M., Xu C., Ji L., Chen Z. (2021). Construction of S-scheme 0D/2D heterostructures for enhanced visible-light-driven CO_2_ reduction. Appl. Catal. B Environ..

[27] Stone D., Gigi S., Naor T., Li X., Banin U. (2024). Size-dependent photocatalysis by wurtzite InP quantum dots utilizing the red spectral region. ACS Energy Lett..

[28] Wang H., Zhao R., Hu H., Fan X., Zhang D., Wang D. (2020). 0D/2D heterojunctions of Ti_3_C_2_ MXene QDs/SiC as an efficient and robust photocatalyst for boosting the visible photocatalytic NO pollutant removal ability. ACS Appl. Mater. Interfaces.

[29] Wang Z., Al Jitan S., AlNashef I., Tardy B.L., Palmisano G. (2024). Recent progress of MXene as a cocatalyst in photocatalytic carbon dioxide reduction. Chem. Eng. J. Adv..

[30] Lai C., An Z., Yi H., Huo X., Qin L., Liu X., Li B., Zhang M., Liu S., Li L. (2021). Enhanced visible-light-driven photocatalytic activity of bismuth oxide via the decoration of titanium carbide quantum dots. J. Colloid Interface Sci..

[31] Lv K., Yang Q., Yan X., Liang L., Liu F., Wang M., Yao H., Wei D., Ma D., Xie K. (2022). Photosensitive Ti_3_C_2_ for dyes degradation. Results Mater..

[32] Yao Z., Sun H., Xiao S., Hu Y., Liu X., Zhang Y. (2022). Ti_3_C_2_ quantum dots modified on BiOBr surface for sewage disposal: The induction of the piezo-phototronic effect from edge to whole. Appl. Surf. Sci..

[33] Saravanakumar K., Yun K., Maheskumar V., Yea Y., Jagan G., Park C.M. (2023). Construction of novel In_2_S_3_/Ti_3_C_2_ MXene quantum dots/SmFeO_3_ Z-scheme heterojunctions for efficient photocatalytic removal of sulfamethoxazole and 4-chlorophenol: Degradation pathways and mechanism insights. Chem. Eng. J..

[34] Wang Q., Zhu F., Cheng H., Komarneni S., Ma J. (2023). Efficient activation of persulfate by Ti_3_C_2_ MXene QDs modified ZnFe_2_O_4_ for the rapid degradation of tetracycline. Chemosphere.

[35] Yin H., Pu B., Jiang H., He H., Han T., Wang W., Yu C., Wang Z., Li X. (2024). Highly Active MXene quantum dots/CuSe n-p plasmonic heterostructures for ultrafast photocatalytic removal of Cr(VI) under full solar spectrum. Langmuir.

[36] Zhang H., Tian L., Zhang Z., Han J., Wu Z., Wei Z., Wang S., Cao Y., Zhang S., Zhang Y. (2024). Effective degradation of phenol by in-situ photocatalytic-Fenton-like technology with BiVO_4_/Bi_2_WO_6_/Ti_3_C_2_ QDs. Surf. Interfaces.

[37] Miao Z., Wang G., Zhang X., Dong X. (2020). Oxygen vacancies modified TiO_2_/Ti_3_C_2_ derived from MXenes for enhanced photocatalytic degradation of organic pollutants: The crucial role of oxygen vacancy to schottky junction. Appl. Surf. Sci..

[38] Wang A., Wen Y., Zhu H., Liu Z., Wang H., Yao W., Fan Y., Xie G., Chen X., Yan K. (2024). Oxygen vacancy mediated photocatalysis of Ti_3_C_2_ MXene quantum dots/W_18_O_49_ hybrid membrane for peroxymonosulfate-enhanced oxidation degradation. Chem. Eng. J..

[39] Cheng T., Xing Z., Zhang N., Sun P., Peng H., Li Z., Wang N., Zhou W. (2024). Ti_3_C_2_ quantum dots-modified oxygen-vacancy-rich BiOBr hollow microspheres toward optimized photocatalytic performance. Chemosphere.

[40] Qi N., Mo L., Tang Y., He Y., Wang Y., Shi X., Fang Y., Liu X., Wei S. (2025). The Schottky junction between Ti_3_C_2_ MXene quantum dots and Bi_2_WO_6_ with oxygen vacancies accelerates charge separation and improves the photocatalytic degradation efficiency of tetracycline. J. Environ. Chem. Eng..

[41] Yao Z., Zuo L., Yang H., Qin B., Li X., Wang J., Wang F. (2025). Ti_3_C_2_ quantum dots/hydroxypropyl methylcellulose modified Bi_2_WO_6_ for enhancing piezo-photocatalytic degradation of tetracycline. J. Colloid Interface Sci..

[42] Li Q., Zhu J., Zhao T., Fang M., Chen N., Hou G., Zhao X., Tang Z., Wu F. (2026). Bioinspired petal-architected 0D/2D Ti_3_C_2_ QDs @ Bi_2_WO_6_ heterojunctions with quantum dot confinement for enhanced photocatalytic ibuprofen degradation. Sep. Purif. Technol..

[43] Shi Q., Huang S., Xi Q., Niu Y., Wen N., Du L., Lv Y., Peng T. (2025). Preparation and photocatalytic degradation of PTCN/SnS_2_/Ti_3_C_2_ QDs composites. J. Indian Chem. Soc..

[44] Fattah-alhosseini A., Sangarimotlagh Z., Karbasi M., Duygulu O., Dikici B., Kaseem M. (2026). Ti_3_C_2_-MXene quantum dot/Fe-MOF heterostructure with enhanced visible-light photocatalytic performance. Adv. Compos. Hybrid Mater..

[45] Du X., Zhao T., Xiu Z., Xing Z., Li Z., Pan K., Yang S., Zhou W. (2020). BiVO_4_@ZnIn_2_S_4_/Ti_3_C_2_ MXene quantum dots assembly all-solid-state direct Z-Scheme photocatalysts for efficient visible-light-driven overall water splitting. Appl. Mater. Today.

[46] Yang L., Chen Z., Wang X., Jin M. (2022). High-stability Ti_3_C_2_-QDs/ZnIn_2_S_4_/Ti(IV) flower-like heterojunction for boosted photocatalytic hydrogen evolution. Nanomaterials.

[47] Honda M., Yoshii Y., Okayama N., Ichikawa Y. (2024). Low Temperature Synthesis of 3d Metal (Fe, Co, Ni, Cu)-Doped TiO_2_ photocatalyst via liquid phase deposition technique. Sustain. Chem..

[48] Ding L., Zeng S., Zhang W., Guo C., Chen X., Peng B., Lv Z., Zhou H., Xu Q. (2022). Nitrogen-Doped Ti_3_C_2_ MXene quantum dots/1D CdS nanorod heterostructure photocatalyst of highly efficient hydrogen evolution. ACS Appl. Energy Mater..

[49] Li Y., Ding L., Guo Y., Liang Z., Cui H., Tian J. (2019). Boosting the photocatalytic ability of g-C_3_N_4_ for hydrogen production by Ti_3_C_2_ MXene quantum dots. ACS Appl. Mater. Interfaces.

[50] Dong G., Zhang Y., Wang Y., Deng Q., Qin C., Hu Y., Zhou Y., Tian G. (2021). Ti_3_C_2_ quantum dots modified 3D/2D TiO_2_/g-C_3_N_4_ S-scheme heterostructures for highly efficient photocatalytic hydrogen evolution. ACS Appl. Energy Mater..

[51] Camgöz B., Demi E., Akyüz D. (2026). Enhancement of photocatalytic hydrogen production with CoP/g-C_3_N_4_/Ti_3_C_2_ quantum dots. Int. J. Hydrogen Energy.

[52] Hamdan S.W., Yusuf A.O., Othman I., Al Jitan S., Helal M.I., Žerjav G., Pintar A., Vega L.F., Al-Ali K., Palmisano G. (2026). Ti_3_C_2_ MXene quantum dots-modified 2D/3D Ni_2_P/Zn_0.5_Cd_0.5_S heterostructures for efficient solar-driven hydrogen evolution. J. Environ. Chem. Eng..

[53] Wang Y., Li X., Sun L., Yuan H., Sun X. (2026). Efficient piezo-photocatalytic production of hydrogen by Ti_3_C_2_ MXene quantum dots modified BaTiO_3_ nanowires. J. Photochem. Photobiol. A Chem..

[54] Tang R., Zhou S., Li C., Chen R., Zhang L., Zhang Z., Yin L. (2020). Janus-Structured Co- Ti_3_C_2_ MXene quantum dots as a schottky catalyst for high-performance photoelectrochemical water oxidation. Adv. Funct. Mater..

[55] Chen X., Xu W., Shi Z., Ji Y., Lyu J., Pan G., Zhu J., Tian Y., Li X., Song H. (2021). Plasmonic gold nanorods decorated Ti_3_C_2_ MXene quantum dots-interspersed nanosheet for full-spectrum photoelectrochemical water splitting. Chem. Eng. J..

[56] Song Y., Zhang X., Zhang Y., Zhai P., Li Z., Jin D., Cao J., Wang C., Zhang B., Gao J. (2022). Engineering MoO_x_/MXene hole transfer layers for unexpected boosting of photoelectrochemical water oxidation. Angew. Chem. Int. Ed..

[57] Li Q., Cui X., Liu X., Wang W. (2025). Ti_3_C_2_ quantum dots decorated BiVO_4_ photoelectrode for both photoelectrochemical water splitting and H_2_O_2_ determination. J. Alloys Compd..

